# Extensive Expression Analysis of *Htt* Transcripts in Brain Regions from the zQ175 HD Mouse Model Using a QuantiGene Multiplex Assay

**DOI:** 10.1038/s41598-019-52411-2

**Published:** 2019-11-06

**Authors:** Aikaterini S. Papadopoulou, Casandra Gomez-Paredes, Michael A. Mason, Bridget A. Taxy, David Howland, Gillian P. Bates

**Affiliations:** 1 Huntington’s Disease Centre, Department of Neurodegenerative Disease and UK Dementia Research Institute at UCL, Queen Square Institute of Neurology, University College London, London, WC1N 3BG UK; 2 CHDI Management/CHDI Foundation Inc., New York, NY 10001 USA

**Keywords:** Neuroscience, Diseases of the nervous system, Huntington's disease

## Abstract

Huntington’s disease (HD) is an inherited neurodegenerative disorder caused by a CAG repeat expansion within exon 1 of the huntingtin (*HTT*) gene. *HTT* mRNA contains 67 exons and does not always splice between exon 1 and exon 2 leading to the production of a small polyadenylated *HTTexon*1 transcript, and the full-length *HTT* mRNA has three 3′UTR isoforms. We have developed a QuantiGene multiplex panel for the simultaneous detection of all of these mouse *Htt* transcripts directly from tissue lysates and demonstrate that this can replace the more work-intensive Taqman qPCR assays. We have applied this to the analysis of brain regions from the zQ175 HD mouse model and wild type littermates at two months of age. We show that the incomplete splicing of *Htt* occurs throughout the brain and confirm that this originates from the mutant and not endogenous *Htt* allele. Given that *HTTexon*1 encodes the highly pathogenic exon 1 HTT protein, it is essential that the levels of all *Htt* transcripts can be monitored when evaluating HTT lowering approaches. Our QuantiGene panel will allow the rapid comparative assessment of all *Htt* transcripts in cell lysates and mouse tissues without the need to first extract RNA.

## Introduction

Huntington’s disease (HD) is an autosomal dominant disorder that is characterised by movement disorders, as well as psychiatric and cognitive disturbances^1^. It is caused by the expansion of a CAG repeat in exon 1 of the huntingtin gene (*HTT*), which is translated to a huntingtin protein (HTT) with an abnormally long polyglutamine tract^2^. CAG repeats of 40 or more are fully penetrant, alleles of 36 to 39 result in an increased likelihood that HD will occur within a normal life span and individuals with 35 or less remain unaffected^3^. There is a negative correlation between the age of onset of motor symptoms and the length of the CAG repeat as measured in blood, with expansions of 70 and above resulting in onset before 20 years of age. Somatic expansion of the CAG repeat in neurons can lead to CAG repeat lengths in brain that are considerably longer than those present in blood^4,5^.

The mammalian *HTT* gene is approximately 180 kb in length and contains 67 exons. Two mature transcripts of approximately 10.3 and 13.7 kb were initially described that differed in the length of their 3′UTRs^6^. The recent identification of an additional polyadenylation (poly(A)) site in the 3’UTR results in a *HTT* transcript of approximately 12.5 kb, with the result that we now know that three full-length human *HTT* transcripts are produced^7^. In addition, we have previously shown that exon 1 of *HTT* does not always splice to exon 2, resulting in two small polyadenylated mRNAs (*Httexon*1)^8^. In mouse, these extend to cryptic poly(A) sites located at 680 and 1,145 bp into intron 1^8,9^, and in human, to cryptic poly(A) sites extending to 2,710 and 7,327 bp into intron 1^8,10^. The extent to which this incomplete splicing event occurs increases with increasing CAG repeat length^8^ and the *Httexon1* transcripts are translated to generate the highly pathogenic exon 1 HTT protein^8^. These alternative mRNA processing events mean that at least five transcripts are produced from the human *HTT* and mouse *Htt* genes, which are translated to produce either full length HTT or the exon 1 HTT protein.

Strategies to lower the levels of *HTT* mRNA are a major focus for HD therapeutics and are at various stages of development. These were discussed in a recent review^11^ and include antisense oligonucleotides (ASOs), interfering RNAs (RNAi) and small molecule splicing modulators for targeting RNA, and zinc-finger transcription factors and CRISPR/Cas9 for targeting DNA. ASOs targeting *HTT* mRNA are the most advanced, and their intrathecal delivery has been shown to be tolerated in a phase II clinical trial and to result in the reduction of mutant HTT protein in cerebrospinal fluid (CSF)^12^. In some cases, these strategies target all *HTT* transcripts, but in others, they target the full length transcripts whilst leaving the *Httexon1* transcripts intact. As the exon 1 HTT protein is highly pathogenic^13,14^, and the relative contribution of the mutant forms of the full length HTT and the exon 1 HTT proteins to disease pathogenesis is unknown, it is important that we can measure the extent to which the levels of all *HTT* transcripts are affected by these various strategies.

QuantiGene assays are based on branched DNA (bDNA) technology^15–17^, have a workflow that is easy and fast to perform in a multiplex 96-well plate format, are devoid of additional steps after homogenisation of the tissues of interest and are sensitive enough to detect low abundance transcripts. QuantiGene singleplex assays have been used in the past for quantifying huntingtin mRNA expression^18–20^. Therefore, we set out to establish a QuantiGene multiplex assay to provide a method for measuring all of the mouse *Htt* transcripts of interest concomitantly and validated this through the direct comparison with quantitative real-time PCR (qPCR). We applied this assay to measure *Htt* transcripts in the zQ175 knock-in model of HD^21,22^ that is used extensively for therapeutic target validation. We extended previous work^8^ to show that the *Httexon1* transcript is present throughout the brain in this model and provide further evidence to confirm that it originates from the mutant *Htt* allele. In summary, the QuantiGene multiplex assay provides a rapid and reliable means of measuring the levels of mouse *Htt* transcripts directly in tissue homogenates.

## Results

### QuantiGene *Htt* multiplex assay custom design

**Figure 1:**
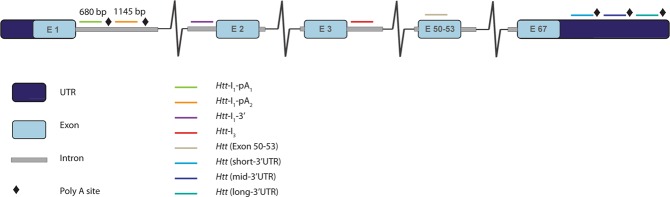
Schematic representation of the location of the probe sets for the multiplex *Htt* QuantiGene assays. Custom probe sets were designed against *Htt* mRNA sequence to enable the measurement of all major *Htt* transcripts. The 9-plex assay contained probe sets to intron 1 sequences before the first cryptic polyA site at 680 bp to detect the *Httexon*1 transcripts, to the 3′ end of intron 1 and to intron 3 as controls for unprocessed mRNA, and to exons 50–53 for all *Htt-FL* transcripts. The 10-plex assay included a probe set to sequences between the first and second cryptic polyA sites, between 680 and 1145 bp. The 14-plex assay also included probe sets to each of the three 3′UTRs of the full-length transcript.

In collaboration with Thermofisher, we set out to design a multiplex QuantiGene panel to detect the incompletely spliced (*Httexon1*) and full-length *Htt* (*Htt-FL*) transcripts (Fig. 1). This contained probes to intron 1 before the first cryptic poly(A) site at 680 bp (*I*_1_*-pA*_1_), to intron 1 sequences after this and before the second cryptic poly(A) site at 1,145 bp (*I*_1_*–pA*_2_), to sequences at the 3′ end of intron 1 (*I*_1_*-*3′), within intron 3 (*I*_3_) and to exons 50–53 for the fully-spliced coding sequence (E50-53). The two cryptic poly(A) sites are 465 bp apart, raising the possibility that the amplification trees corresponding to these two sequences might interfere due to their close proximity. To address this, we designed the 9-plex and 10-plex QuantiGene panels outlined in Table 1.

**Table 1:** Design of the 9-plex and 10-plex QuantiGene assays for the detection of *Htt* transcripts.

Transcript/gene symbol	Transcript/gene name	GOI/HK	9-plex	10-plex
*(Htt)-I*_*1*_-*pA*_*1*_	*Huntingtin – Intron 1- poly*(*A*)_*1*_	*Htt*	√	√
*(Htt)-I*_*1*_-*pA*_*2*_	*Huntingtin – Intron 1- poly*(*A*)_2_	*Htt*	X	√
*(Htt)- I*_*1*_-*3*’	*Huntingtin – Intron 1–3’*	*Htt*	√	√
*(Htt-)-I*_*3*_	*Huntingtin – Intron 3*	*Htt*	√	√
*Htt-FL (E50-53)*	*Huntingtin – exons 50–53*	*Htt*	√	√
*Eif4a2*	*Eukaryotic Initiation factor 4a2*	*HK*	√	√
*Rpl13a*	*Ribosomal protein L 13a*	*HK*	√	√
*Ubc*	*Ubiquitin C*	*HK*	√	√
*CanX*	*Calnexin*	*HK*	√	√
*Atp5b*	*ATP synthase subunit beta*	*HK*	√	√

GOI = gene of interest, HK = housekeeping reference gene.

The *I*_1_*-pA*_1_ probe set identifies the *Httexon1* transcripts that terminate at both the first and the second cryptic poly(A) signals. The *I*_1_*-pA*_2_ probe set recognises only the *Httexon1* transcript that terminates at the second poly(A) signal, while the *I*_1_*-*3′ probe set would identify incompletely spliced intron 1 sequences that have not terminated at cryptic poly(A) signals. The *I*_3_ probe set serves to control for any contaminating *Htt* pre-mRNA. The housekeeping reference genes chosen for the panels have been identified previously for their robustness and stability when used in brain tissues from HD mouse models^23^.

### Establishment and optimisation of QuantiGene *Htt* multiplex assays

To examine the linearity of the probe set signals for each brain region, we used a pool of equimolar lysate from wild type (WT) and zQ175 heterozygous animals (n = 4/genotype). The two pools were then subjected to 2-fold serial dilutions and QuantiGene analysis using our 9-plex and 10-plex panels.

**Figure 2:**
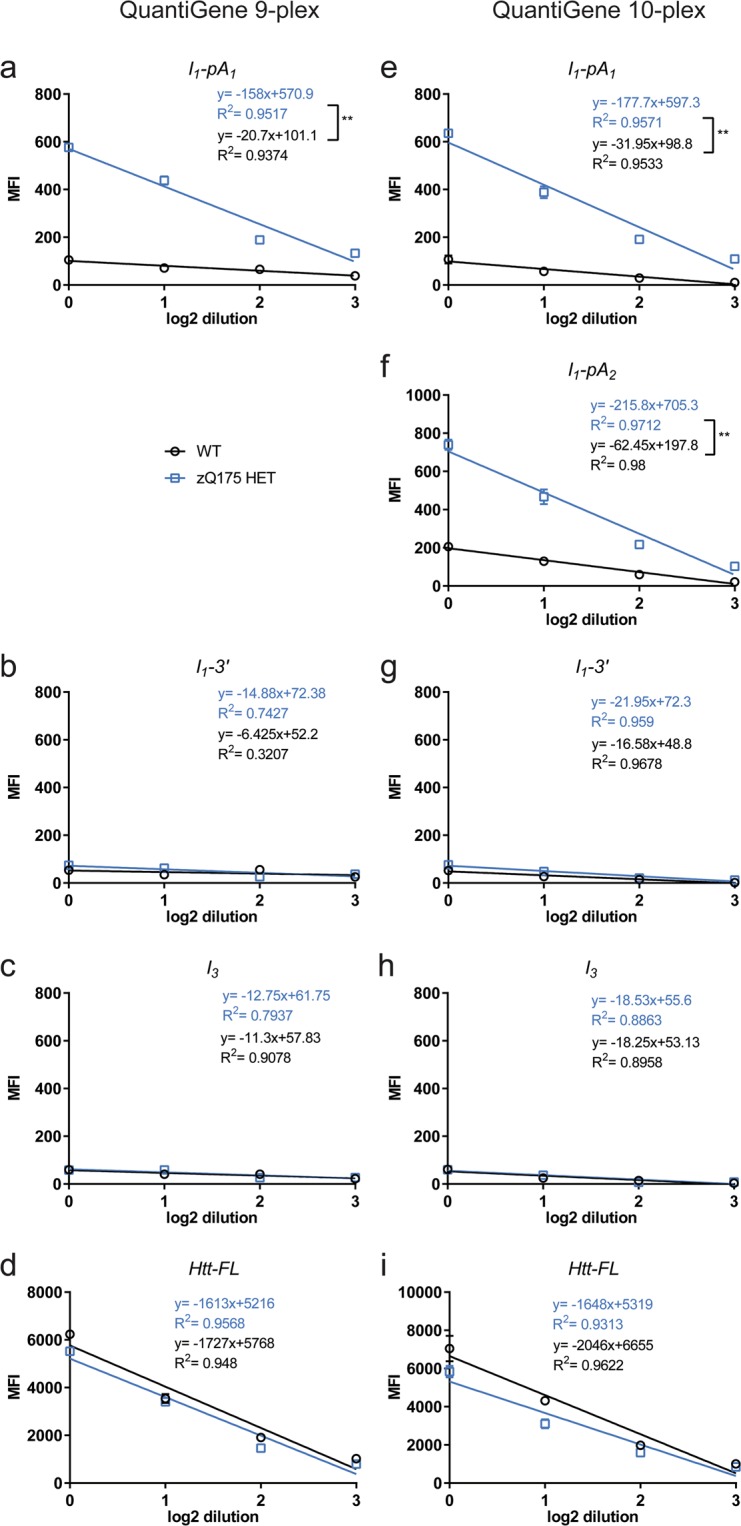
Establishment and optimisation of QuantiGene *Htt* multiplex assays in striatal tissue from WT and zQ175 heterozygous mice. The MFI signals corresponding to 2-fold serial dilutions of the QuantiGene striatal lysate from 2 month old animals are shown for each *Htt* transcript probe set. (**a**–**d**) 9-plex QuantiGene assay and (**e–i**) 10-plex QuantiGene assay. (**a**,**e**,**f**) The linear regression slopes of the *Httexon1* transcript (*I*_1_-*pA*_1_ and *I*_1_–*pA*_2_) are significantly different between the two genotypes, with the WT MFI signal averaging background levels, as measured by the probe set for *Htt I*_3_. (**b**,**g**) The *I*_1_*-3*′ probe set showed that the un-spliced intron 1 sequences do not extend to the 3′ end of intron 1, with a signal similar to that observed for the assay background control, *Htt I*_*3*_. (**c**,**h**). The *I*_3_ probe set served as a control to indicate the level of unprocessed *Htt* mRNA. (**d,i**) *Htt-FL* probe set signals are shown. Data were from two technical replicates of equimolar pools of striatal samples (n = 4/genotype). Statistical analysis was by linear regression **p < 0.01. MFI = mean fluorescence intensity.

The lowest mean fluorescence intensity (MFI) signal for the striatum corresponded to the control *I*_3_ probe set, while the highest corresponded to that for the reference gene *Ubc* (Fig. 2c,h and Supplementary Fig. S1). The MFI signal for the *I*_1_*–*3′ probe set was comparable to the assay background (*I*_3_) even at the highest concentration used (Fig. 2b,g,c,h). The linear regression of the MFI signals for the reference genes *Ubc* and *Eif4a2* in the striatum, for both the 9-plex and 10-plex QuantiGene panels, showed that the signal was saturated for the undiluted material (Supplementary Fig. S1). Thus, the undiluted material was excluded from the linear range for all probe sets in all other brain regions (Supplementary Figs S2–S5). Based on the linearity of the probe set signals and the coefficient of variance (% CV) we recommend a dilution range of the starting material of 1:3–1:4 and never more than 1:5 for all brain regions (Supplementary Table 1). Close evaluation of the *Ubc* signal suggested that saturation had been reached for all brain regions, even at the lowest dilution range, and therefore, *Ubc* was excluded from all further analyses.

Importantly, the striatal MFI signal for the *I*_1_*-pA*_1_ probe set (for both plex panels) (Fig. 2a,e) and for the *I*_1_*-pA*_2_ probe set (for the 10-plex panel) (Fig. 2f) suggested that the genotypes behaved differently for these early intron 1 sequences. There was considerably more signal in the zQ175 pool than in the WT, as indicated by the difference in the linear regression slopes. The MFI signal for the *I*_1_*-pA*_1_ probe set showed a similar linear range of detection in both the 9- and 10-plex panels (Fig. 2a,e). The signal for the *I*_1_*-pA*_2_ probe set on the 10-plex panel (Fig. 2f) was significantly greater for the zQ175 heterozygotes than for WT. The MFI signal for the *I*_1_*-*3′ probe sets was comparable to *I*_3_ for both WT and zQ175 lysates (Fig. 2b,g,c,h for striatum and Supplementary Figs S2–S5 for other brain regions). The linear ranges for the probe sets for *Htt-FL* are shown in Fig. 2d,i, and Supplementary Figs S2–S5. Taken together, these data suggest that the 10-plex QuantiGene panel can be used to detect all *Htt* transcripts, without compromising the fluorescent signal due to the close proximity between the *I*_1_*-pA*_1_ and *I*_1_*-pA*_2_ probe sets. At the dilutions recommended, the housekeeping genes gave comparable signals for the two genotypes (Supplementary Figs S1–S5).

### Validation of the QuantiGene *Htt* 10-plex assay

We set out to validate our 10-plex QuantiGene panel by comparing the expression levels of the *Htt* transcripts as measured by this approach with that determined by real time quantitative PCR (qPCR). The levels of *Htt* transcripts were measured in brain regions from zQ175 heterozygotes and WT littermates at 2 months of age (n = 4/genotype). The locations of the qPCR assays corresponded to the position of the QuantiGene probe sets, except that we did not include a qPCR assay for intron 3 (Supplementary Table S2).

**Figure 3:**
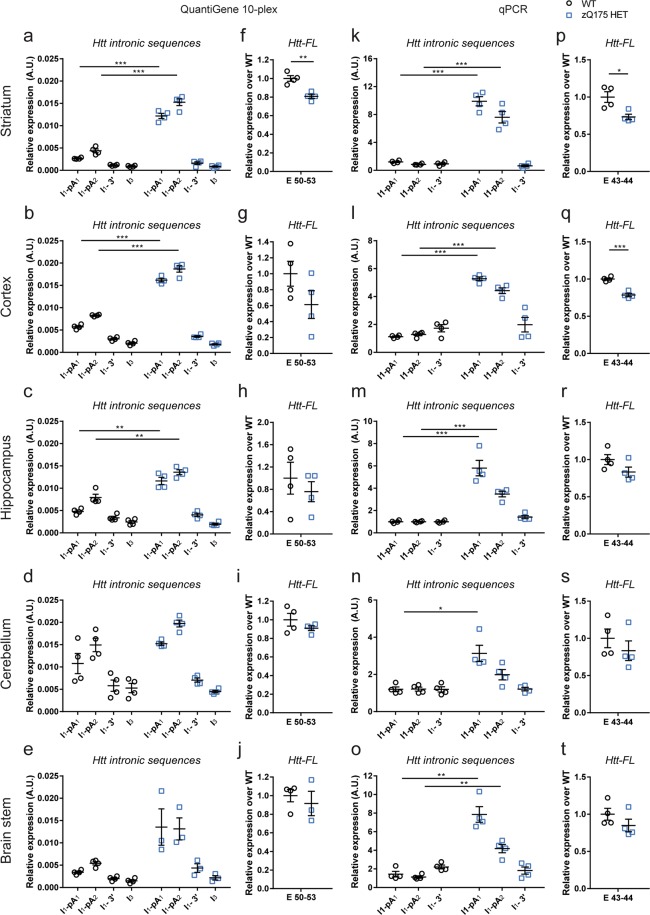
Comparison of the analysis of *Htt* transcripts in the striatum of zQ175 heterozygous animals using the 10-plex QuantiGene panel and qPCR assays. Expression of the *Htt* intronic sequences and *Htt-FL* transcripts was measured by our QuantiGene 10-plex assay (**a–j**) and qPCR (k-t) in striatum, cortex, hippocampus, cerebellum and brain stem of 2 month old mice, and presented relative to that for housekeeping genes. (**a–e** and **k–o**) *Httexon1* transcripts can be detected in all brain regions by both approaches. (**f–j** and **p–t**) The levels of *Htt-FL* as measured by both approaches were comparable. Data were obtained from n = 4/genotype with two technical replicates for QuantiGene and three for qPCRs. Statistical analysis was by multiple *t*-test with Benjamini-Hochberg with an alpha of 0.05; *p < 0.05, **p < 0.01, ***p < 0.001. Tissue lysate dilutions were 1:3 for striatum and brain stem, 1:4 for cortex and cerebellum and 1:5 for hippocampus.

The level of the *Httexon1* transcript was shown to be abundant in the striatum, cortex, hippocampus, cerebellum and brain stem of zQ175 mice as compared to WT by both approaches (Fig. 3). Both assays confirm that the level of 3′ intron 1 sequences are at background levels (Fig. 3). This is in keeping with our previous RNAseq data indicating that transcription terminates at the second cryptic poly(A) site^8^. In the zQ175 samples, the signal from the QuantiGene *I*_1_*-pA*_2_ probe set is higher than that for *I*_1_*-pA*_1_ (Fig. 3a-d). As it is not possible for the level of the intron 1 sequences between poly(A)_1_ and poly(A)_2_ to be greater than that before poly(A)_1_, this may reflect differences in hybridisation efficiencies. This most likely accounts for the higher *I*_1_*-pA*_2_ signal in WT mice, which we consider to be background noise; the qPCR data suggest that any level of incomplete splicing that occurs in WT mice is below the level of detection (the readings for the *I*_1_*-pA*_1_ and *I*_1_*-pA*_2_ assays are not greater than for *I*_1_*-3*′).

The *Htt-FL* transcript was also analysed in all five brain regions. In the striatum, the level of *Htt-FL* was reduced to approximately 70% – 80% of WT levels in the zQ175 mice as measured by both assays (Fig. 3f,p). For all of the other brain regions, the situation was less clear cut, although in all cases the mean level of *Htt-FL* expression was lower in the zQ175 than WT mice (Fig. 3). The qPCR assay gave tighter data for the cortex in which the reduction of *Htt-FL* could be detected (Fig. 3q). In comparison, the QuantiGene data was more variable, and so we would recommend that more than 4 samples per genotype should be analysed.

### Design and application of a QuantiGene *Htt* 14-plex assay

We next extended our 10-plex assay to include probes against each of the three 3′UTR isoforms of *Htt* (short-, mid- and long- 3′UTR)^7^ and an additional housekeeping reference gene, *Ppib*^18,20^ (Fig. 1 and Supplementary Table S3). To ensure that the new 14-plex panel could detect all *Htt* transcripts and *Ppib*, we analysed the same zQ175 and WT lysates as had been used for the 10-plex panel (n = 4/genotype). The *Httexon1* transcript levels in zQ175 mice were comparable to those detected with the 10-plex assay (Supplementary Fig. S6 and Fig. 3), except in brain stem, where they showed less variability, and consequently, the difference in the levels between zQ175 and WT samples was statistically significant. The E50-53 probe set, as well as each of the 3′UTR assays, showed a statistically significant reduction in *Htt-FL* levels in zQ175 as compared to WT samples for the striatum, cortex and hippocampus (Supplementary Fig. 6).

**Figure 4:**
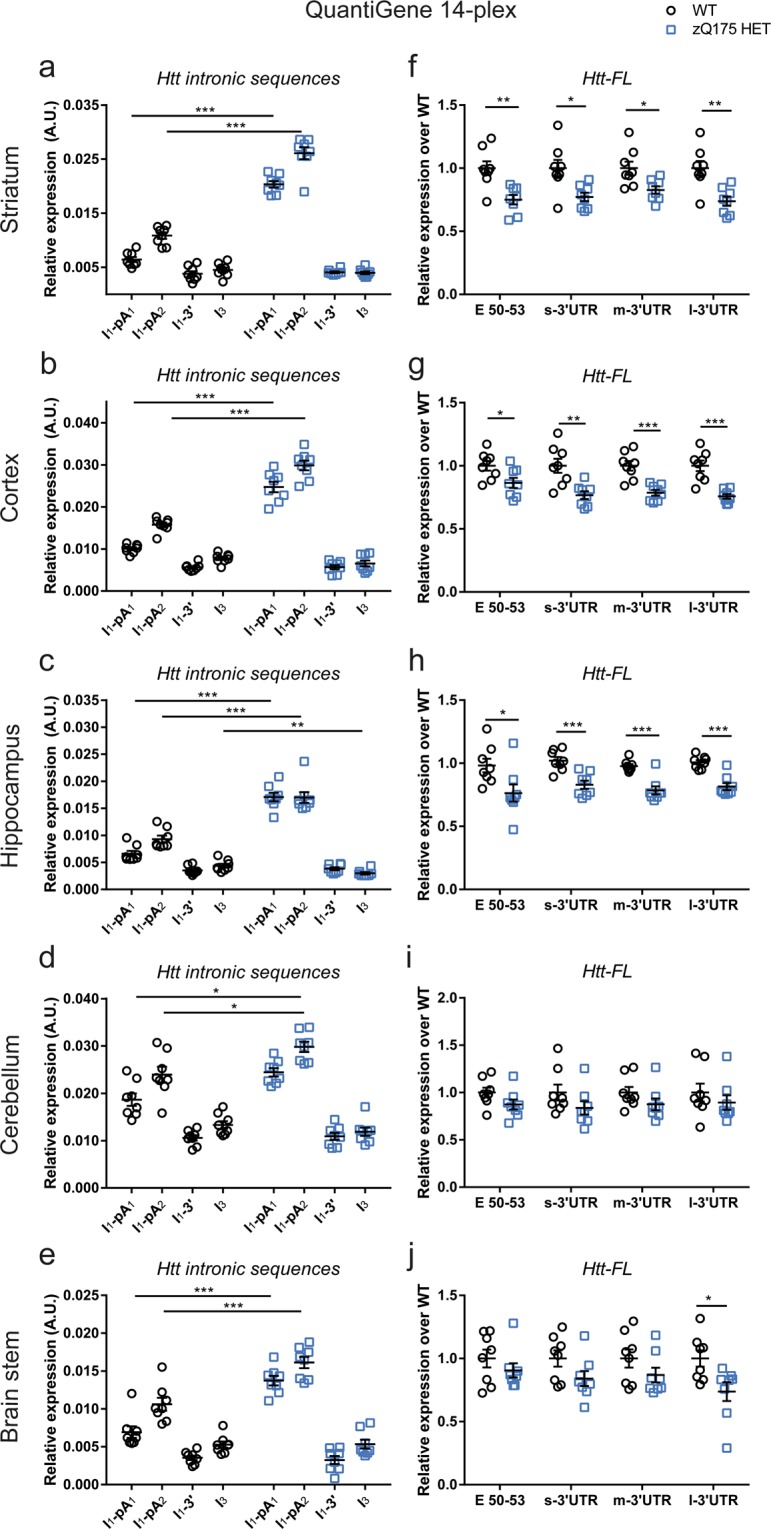
Expression analysis of *Htt* transcripts in five brain regions using the custom-made 14-plex QuantiGene assay. The expression of intronic *Htt* transcripts in 2 month old mice are presented relative to that for housekeeping genes. (**a–e**) The *Httexon1* transcript is present in all zQ175 brain regions. (**f–j**) The level of the *Htt-FL* transcript is significantly decreased in the striatum, cortex and hippocampus as measured by the E50-53 and all 3′UTR probe sets. Data were from n = 4/genotype with two technical replicates. Statistical analysis was by multiple *t*-test with Benjamini-Hochberg with an alpha of 0.05, *p < 0.05, **p < 0.01, ***p < 0.001. Tissue lysate dilutions were 1:3 for striatum, hippocampus and brain stem, 1:4 for cortex and cerebellum.

To further assess the application of the 14-plex QuantiGene panel, and to test the consistency of our data, we repeated our analysis of *Htt* transcripts in a different cohort of mice (n = 8/genotype) (Fig. 4). We could detect a statistically significant increase in the *Httexon1* transcript in the zQ175 mice as compared to WT in all five brain regions tested (Fig. 4a-e). This correlated with a statistically significant reduction in *Htt*-*FL* levels as measured by all four of the *Htt-FL* assays for striatum (~23%), cortex (~22%) and hippocampus (~22%) (Fig. 4f-j).

Overall, we conclude that our 14-plex QuantiGene assay can reliably compare differences in the levels of the *Httexon1* and *Htt-FL* transcripts between the zQ175 mouse model of HD and WT littermates.

### The level of the *Httexon1* transcript in *z*Q175 tissues corresponds to a reduction in mutant and not endogenous *Htt-FL*

**Figure 5:**
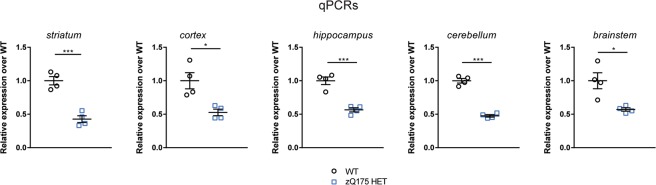
The level of the endogenous *Htt*-*FL* transcript is not decreased in zQ175 tissues. Allele-specific qPCR analysis of the level of the endogenous *Htt-FL* transcript in five brain regions from 2 month old WT and zQ175 mice. The level of the endogenous *Htt-FL* transcript was present at 50% WT levels in the zQ175 samples for all five brain regions. Data were obtained from n = 4/genotype. Statistical analysis was by *t*-test, *p < 0.05, ***p < 0.001.

We found that the level of the *Htt*-*FL* transcript was consistently decreased in zQ175 striatum, cortex and hippocampus (Fig. 4) and Supplementary Fig. S6). In order to determine whether this reflected a decrease in the endogenous transcript, the mutant transcript, or both, we employed an allele-specific qPCR assay (Fig. 5). We found that the level of endogenous *Htt-FL* was 50% of WT levels in all five brain regions for zQ175 mice. Therefore, any reductions in the level of *Htt-FL* must correspond to decreases in the mutant *Htt-FL* transcript. This is consistent with our failure to detect incomplete splicing of the endogenous *Htt* transcript in WT mice (Figs 2 and 4 and Sathasivam *et al*. 2013).

## Discussion

In this study, we have assessed the expression of all known major *Htt* transcripts in the brains of the zQ175 mouse model of HD. We developed a multiplex QuantiGene assay based on the rapid, robust and high-throughput bDNA technology^17^ for this purpose and demonstrated that this can be used to replace qPCR for studies comparing *Htt* transcripts between samples. We applied the QuantiGene assay to the comparison of *Htt* transcripts in various brain regions between zQ175 and WT littermates at 2 months of age, and showed that incomplete slicing occurs throughout the brain for zQ175, but not WT, animals. Finally, we provide evidence to indicate that the *Httexon1* transcript originates from the incomplete splicing of the mutant *Htt* allele.

We have developed and optimised a 14-plex QuantiGene assay to enable us to detect all major *Htt* transcripts directly from tissue lysates, obviating the need to prepare RNA and replacing the more labour-intensive qPCR. This assay includes probe sets targeting sequences extending to the first and second cryptic poly(A) sites in intron 1, to detect incomplete splicing; to the 3′ end of intron 1 and to intron 3, to give an indication of pre-mRNA background; to exons 50–53 and each of the 3′UTRs (short, mid and long) to detect *Htt-FL* and shifts in 3′UTR usage; as well as six housekeeping genes. We have applied this to the analysis of *Htt* transcripts in five brain regions from the zQ175 knock-in mouse model of HD and WT littermates. For each brain region, we calculated the lysate dilution required to ensure that mean fluorescence intensity (MFI) signals were in the linear range and recommend using dilutions at the lower end of the range detailed in Supplementary Table S1. We caution against using lysates that have been subjected to multiple freeze-thaw cycles, and a recent study suggested that the use of ‘RNAlater’, when preparing QuantiGene lysates, might better preserve long mRNAs, such as *Htt-FL*, which are more prone to degradation^18^.

QuantiGene analysis of *Htt* transcripts in brain regions of 2 month old zQ175 mice (Fig. 4) showed that intronic sequences 5′ to the cryptic poly(A) sites (*I*_1_*-pA*_1_ and *I*_1_*-pA*_2_) were present in zQ175 cortex, striatum, hippocampus, cerebellum and brain stem at higher levels than in WT, indicating that incomplete splicing of *Htt* occurs in all of these brain regions. The levels of intronic sequences at the 3′ end of intron 1 (*I*_1_*-*3′) were comparable to intron 3 (*I*_3_), indicative of pre-mRNA background. This is consistent with our previous RNAseq data showing that the incompletely spliced intron 1 sequence reads terminate at the second cryptic poly(A) site^8^. In all cases, the level of intron 1 sequences between the two poly(A) sites (*I*_1_*-pA*_2_) was higher than that before the first poly(A) site (*I*_1_*-pA*_1_). As it is not possible that the mRNA level between the poly(A) sites is higher than before the first poly(A), this must be a reflection of the differential efficiency of probe binding. The 14-plex analysis of WT tissues (*I*_1_*-pA*_1_ and *I*_1_*-pA*_2_) suggested that the incomplete splicing of *Htt* might be occurring in WT mice to a lesser extent. However, examination of the qPCR data (Fig. 3), for which the *I*_1_*-pA*_1_, *I*_1_*-pA*_2_ and *I*_1_*-3*′ assays give comparable data, indicates that this is unlikely, but arising instead from the differential efficiency of probe-set binding. Therefore, the 14-plex QuantiGene assay provides a valuable tool for comparative analyses between samples but, as used here, is not quantitative.

The level of *Htt-FL* transcript, as detected by all four probe sets (E50-53, s-3′UTR, m-3′UTR and l-3′UTR) is lower in zQ175 striatum, cortex and hippocampus than in WT mice. We employed an allele specific qPCR assay that selectively detects endogenous *Htt* in this knock-in model, and found that the level of the endogenous *Htt-FL* was not decreased in the heterozygous zQ175 mice. That the reduction in *Htt-FL* corresponds to a decrease in the mutant transcript, and that this is a consequence of incomplete splicing between exon 1 and exon 2 is consistent with our observation that incomplete splicing of endogenous *Htt* does not occur in WT mice and our previous RNAseq data^8^.

We have previously shown that the incomplete splicing of exon 1 to exon 2 of *Htt* results in the *Httexon*1 polyadenylated mRNA that is translated to produce the highly pathogenic exon 1 HTT protein^8–10^. The extent of incomplete splicing increases with increasing CAG repeat length, therefore, somatic CAG expansion would be expected to result in the increased production of the exon 1 HTT protein. Genome-wide association studies have identified a number of DNA repair genes as genetic modifiers of HD that have been shown to modulate somatic expansion levels^24–26^. Therefore, incomplete splicing produces a direct link between the genetic modifiers for HD and the production of a pathogenic fragment of HTT. As *Htt* lowering approaches are evaluated in mouse models, it is essential that their effect on the level of *Httexon*1 as well as *Htt-FL* transcripts can be assessed. Our QuantiGene assay provides a rapid method for screening *Htt* lowering agents targeting mouse *Htt* in cell culture and mouse models of HD.

## Methods

### Animal colony maintenance and breeding

All animal care and procedures were performed under a Home Office License in compliance with the Animals and Scientific Procedures Act, 1996, with approval by the University College London Ethical Review Process Committee. The zQ175 (delta neo)^27^ heterozygous colony^21,22^ was maintained by backcrossing to C57BL/6 J (Charles River). The animals had unconditional access to food and water, environmental enrichment in the form of two small aspen bricks and a play tunnel (Datesand Group) and were subjected to a 12-hour light/dark cycle. Animals were sacrificed at 2 months of age, brains dissected, frozen immediately in liquid nitrogen and stored at −80 °C.

### DNA extractions, genotyping and repeat sizing

Genomic DNA isolation was performed after lysing an ear-punch in 50 mM Tris-HCl, 0.5% SDS, 100 mM EDTA pH = 8.0, with 0.1 mg/mL proteinase K at 50 °C overnight. Addition of 300 μL saturated 36% (w/v) NaCl solution was followed by thorough mixing and centrifugation at 21,000 *g* for 30 min. The DNA-containing supernatant was transferred to a new tube with 650 μL ice-cold 100% ethanol (EtOH) followed by mixing and centrifugation at 21,000 *g* for 20 min. The DNA-containing pellet was washed with 70% EtOH and centrifuged at 21,000 *g* for 15 min. The supernatant was discarded, the DNA pellet was left to air dry and lastly the DNA was resuspended in 60 μL 5 mM Tris-HCl, pH = 8.0. Genotyping of the zQ175 animals was performed as follows: in a total volume of 20 μL reaction, 20 ng of DNA was added to a mixture of 1x Go Taq Flexi buffer (Promega), 2.5 mM MgCl_2_, 0.2 mM dNTPs, 1 μM each primer (Forward: 5′-AGGAGCCGCTGCACCGA-3′ and Reverse: 5′-CTCTTCACAACAGTCATGTGCG-3′) and 0.05 U/ μl GoTaq_2_, and the following PCR programme was used: 98 °C for 30 s, followed by 34 cycles of 98 °C for 15 s, 64 °C for 15 s, 72 °C for 30 s, and a final elongation step at 72 °C for 5 min. The PCR products were loaded on a 2.5% agarose gel containing SYBR Safe DNA gel stain (Thermofisher), subjected to electrophoresis and visualised on a BioRad Gel Doc XR+ . The CAG repeat sizing was performed as previously described^28^ using the following primers: Forward: 5′-6FAM-ATGAAGGCCTTCGAGTCCCTCAAGTCCTTC-3′ and Reverse: 5′-GGCGGCTGAGGAAGCTGAGGA-3′, except that the DNA size standard was MapMarker 1000 ROX (BioVentures Inc.) and analysis of data was performed using the GeneMapper plate manager application. The CAG repeat size for the first cohort of the zQ175 mice was 197.75 ± 1.7 (S.D.) and for the second cohort was 207.5 ± 3.02.

### Tissue lysis and QuantiGene assays

All Quantigene probe sets are available from ThermoFisher and the individual accession numbers and probe set regions are summarised in Supplementary Table S3; the sequences for the capture extenders, label extenders and blocking probes are available from ThermoFisher on request. The catalogue number for the 14-plex set validated in this manuscript is 21917. Investigators can select probe sets from this to customise their own multiplex assays, if they did not wish to include all of the *Htt* probe sets or all of the six housekeeping genes.

All QuantiGene reagents were from ThermoFisher. Homogenates of brain regions were prepared by adding 300 μL of working homogenisation solution (homogenisation solution (QG0517) with 1:100 (v/v) 50 mg/ mL Proteinase K (QS0512) for each 10 mg tissue) and using syringes with needles of 23 G and 18 G. Homogenates were then centrifuged at 15,000 *g* for 10 min and the supernatant was transferred to a fresh tube. For linearity probe set assessment, equal volumes of homogenates of the same genotype were pooled together and a 2-fold serial dilution was conducted for each pool. Otherwise, the homogenates were diluted so readings would fit in the linear range of the fluorescent signal for all probe sets in the QuantiGene panel (Supplementary Table S1). The QuantiGene assays were performed as indicated in the manufacturer’s protocol with the exception that the Streptavidin *R*-Phycoerythrin conjugate (SAPE) binding was done at 51 °C. Plates were read using a Magpix (Luminex). Data analysis was performed by normalising the signals obtained for the genes of interest to the geometric mean of the reference gene signals (*Atp5b*, *Eif4a2*, *Rpl*1*3a*, *Canx* and *Ppib* as appropriate). All *Htt-FL* isoforms were expressed relative to the average expression of the WT animals. Technical duplicates of each sample were included in each experiment, with a minimum of two independent experiments for each cohort of animals.

### RNA isolation and cDNA synthesis

Total RNA isolation was conducted using the RNeasy mini kit (Qiagen) following the manufacturer’s protocol, after homogenising the tissue in Qiazol lysis reagent (Qiagen) using syringes with needles of 23 G and 18 G. DNase I treatment was performed in-column using the RNA-free DNase kit (Qiagen) before the RNA was eluted in the appropriate amount of nuclease-free H_2_O (Sigma-Aldrich). The quality and quantity of RNA was measured using a NanoDrop 1000. Reverse transcription was performed on 0.5–1 μg of RNA using the M-MLV reverse transcriptase (RT) (Invitrogen) according to the company’s protocol and using oligo-dT_(18)_ primers (Invitrogen). A negative control was included for each brain region in which no reverse transcriptase was added (-RT).

### Real-time quantitative PCR assays

Real-time quantitative PCR (qPCR) was performed using hydrolysis probes with a single or double quencher (Supplementary Table S2). The reference gene assays are commercially available (Thermofisher Scientific) and comply with the MIQE guidelines^29,30^. Amplification efficiencies were evaluated by 4-fold serial dilutions of a pool of equal volumes of 4 cDNAs from different zQ175 heterozygous animals and are provided in Supplementary Table S2. A 1:10 dilution of the cDNA was used to avoid inhibition of the qPCR reaction. After determining the linear range of cDNA input and the amplification efficiency of the primer sets, a 1:50 dilution of the cDNA was made for all brain regions. Primers and probes for the custom *Htt* assays were from Eurofins and for the allele discrimination assays were from Integrated DNA Technologies. Samples were prepared in 1x TaqMan Fast Advanced Mix (Applied Biosystems), in a final volume of 15 μL, in 96-well thin wall Hard-Shell PCR plates (BioRad). The primer/probe mix for reference genes was used diluted to 1x and for our custom-designed assays, the primers and probes were used at concentrations of 0.3 mM and 0.2 mM respectively. Plates were sealed with Microseal ‘B’ seals (BioRad) and centrifuged at 800 *g* for 30 s. qPCRs were performed in a BioRad CFX96 qPCR machine with the following programme: 95 °C for 40 s, followed by 40 cycles of 95 °C for 7 s and 60 °C for 20 s. For the allele-discrimination assays, the following programme was used: 95 °C for 2 min, followed by 40 cycles of 95 °C for 10 s, 61.8 °C for 30 s, 72 °C for 30 s. Three technical replicates of each biological sample were used for all assays. C*q* values of the triplicates were assessed and values deviating by 0.5 from the mean C*q* value were excluded from further analysis. Normalisation of the data was done either by the 2^−ΔΔCt^ method ^31^ or based on the amplification efficiency of the primer sets^32^ to the geometric mean of all the reference genes (*Eif4a*2, *Atp5b*, *CanX*, *Ubc*, *Rpl1*3*a*). For the allele discrimination qPCR assays data were normalised to the geometric mean of two reference genes (*Eif4a2*, *Atp5b*).

### Statistical analysis

Statistical analysis was performed using multiple two-tailed Student’s *t*-tests followed by Benjamini-Hochberg correction and a desired false discovery rate (*q*) adjusted to 0.05. All statistical analysis was performed using the GraphPad Prism software.

## Supplementary information

Supplementary info

## Data Availability

The datasets generated during and/or analysed during the current study are available from the corresponding author on reasonable request.

## References

[1] Bates GP (2015). Huntington disease. Nat Rev Dis Primers.

[2] The Huntignton’s Disease Collaborative Group (1993). A Novel Gene Containing a Trinucleotide Repeat That Is Expanded and Unstable on Huntingtons-Disease Chromosomes. Cell.

[3] Rubinsztein DC (1996). Phenotypic characterization of individuals with 30-40 CAG repeats in the Huntington disease (HD) gene reveals HD cases with 36 repeats and apparently normal elderly individuals with 36-39 repeats. Am J Hum Genet.

[4] Kennedy L (2003). Dramatic tissue-specific mutation length increases are an early molecular event in Huntington disease pathogenesis. Hum Mol Genet.

[5] Gonitel R (2008). DNA instability in postmitotic neurons. Proc Natl Acad Sci USA.

[6] Lin B (1993). Differential 3′ poly(A)denylation of the Huntington disease gene results in two mRNA species with variable tissue expression. Hum Mol Genet.

[7] Romo L, Ashar-Patel A, Pfister E, Aronin N (2017). Alterations in mRNA 3′ UTR Isoform Abundance Accompany Gene Expression Changes in Human Huntington’s Disease Brains. Cell Rep.

[8] Sathasivam K (2013). Aberrant splicing of HTT generates the pathogenic exon 1 protein in Huntington disease. Proc Natl Acad Sci USA.

[9] Neueder A, Dumas AA, Benjamin AC, Bates GP (2018). Regulatory mechanisms of incomplete huntingtin mRNA splicing. Nat Commun.

[10] Neueder A (2017). The pathogenic exon 1 HTT protein is produced by incomplete splicing in Huntington’s disease patients. Sci Rep.

[11] Tabrizi SJ, Ghosh R, Leavitt BR (2019). Huntingtin Lowering Strategies for Disease Modification in Huntington’s Disease. Neuron.

[12] Tabrizi, S. J. *et al*. Targeting Huntingtin Expression in Patients with Huntington’s Disease. *N Engl J Med*, 10.1056/NEJMoa1900907 (2019). 10.1056/NEJMoa1900907 31059641

[13] Mangiarini L (1996). Exon 1 of the HD gene with an expanded CAG repeat is sufficient to cause a progressive neurological phenotype in transgenic mice. Cell.

[14] Barbaro BA (2015). Comparative study of naturally occurring huntingtin fragments in Drosophila points to exon 1 as the most pathogenic species in Huntington’s disease. Hum Mol Genet.

[15] Canales RD (2006). Evaluation of DNA microarray results with quantitative gene expression platforms. Nat Biotechnol.

[16] Collins ML (1997). A branched DNA signal amplification assay for quantification of nucleic acid targets below 100 molecules/ml. Nucleic Acids Res.

[17] Flagella M (2006). A multiplex branched DNA assay for parallel quantitative gene expression profiling. Anal Biochem.

[18] Coles AH (2016). A High-Throughput Method for Direct Detection of Therapeutic Oligonucleotide-Induced Gene Silencing *In Vivo*. Nucleic Acid Ther.

[19] Alterman, J. F. *et al*. A High-throughput Assay for mRNA Silencing in Primary Cortical Neurons *in vitro* with Oligonucleotide Therapeutics. *Bio Protoc*, **7**, 10.21769/BioProtoc.2501 (2017). 10.21769/BioProtoc.2501 PMC5621760 28966945

[20] Alterman JF (2015). Hydrophobically Modified siRNAs Silence Huntingtin mRNA in Primary Neurons and Mouse Brain. Mol Ther Nucleic Acids.

[21] Heikkinen T (2012). Characterization of neurophysiological and behavioral changes, MRI brain volumetry and 1H MRS in zQ175 knock-in mouse model of Huntington’s disease. PLoS One.

[22] Menalled LB (2012). Comprehensive Behavioral and Molecular Characterization of a New Knock-In Mouse Model of Huntington’s Disease: zQ175. PLoS One.

[23] Benn CL, Fox H, Bates GP (2008). Optimisation of region-specific reference gene selection and relative gene expression analysis methods for pre-clinical trials of Huntington’s disease. Mol Neurodegener.

[24] Consortium G-H (2015). Identification of Genetic Factors that modify clinical onset of Huntington’s disease. Cell.

[25] Goold R (2019). FAN1 modifies Huntington’s disease progression by stabilizing the expanded HTT CAG repeat. Hum Mol Genet.

[26] Moss DJH (2017). Identification of genetic variants associated with Huntington’s disease progression: a genome-wide association study. Lancet Neurol.

[27] Franich, N. R. *et al*. Phenotype onset in Huntington’s disease knock-in mice is correlated with the incomplete splicing of the mutant huntingtin gene. *J Neurosci Res* Epub 10.1002/jnr.24493 (2019). 10.1002/jnr.24493 PMC6801054 31282030

[28] Sathasivam K (2010). Identical oligomeric and fibrillar structures captured from the brains of R6/2 and knock-in mouse models of Huntington’s disease. Hum Mol Genet.

[29] Bustin SA (2010). Why the need for qPCR publication guidelines?–The case for MIQE. Methods.

[30] Bustin SA (2010). MIQE precis: Practical implementation of minimum standard guidelines for fluorescence-based quantitative real-time PCR experiments. BMC Mol Biol.

[31] Livak KJ, Schmittgen TD (2001). Analysis of relative gene expression data using real-time quantitative PCR and the 2(-Delta Delta C(T)) Method. Methods.

[32] Pfaffl MW (2001). A new mathematical model for relative quantification in real-time RT-PCR. Nucleic Acids Res.

