# Supplementary material for: Extensive Expression Analysis of Htt Transcripts in Brain Regions from the zQ175 HD Mouse Model Using a QuantiGene Multiplex Assay
Source: Sci Rep. 2019 Nov 6;9:16137. doi: 10.1038/s41598-019-52411-2 (PMC6834638; doi:10.1038/s41598-019-52411-2)
Supplement: Supplementary file 1 — Supplementary info [file 41598_2019_52411_MOESM1_ESM.pdf]

Extensive Expression Analysis of *Htt* Transcripts in Brain Regions from the zQ175 HD Mouse Model Using a QuantiGene Multiplex Assay

Aikaterini S. Papadopoulou[1], Casandra Gomez-Paredes[1], Michael A. Mason[1],

Bridget A. Taxy[1], David Howland[2] and Gillian P. Bates[1]*

[1]Huntington's Disease Centre, Department of Neurodegenerative Disease and UK Dementia Research Institute at UCL, Queen Square Institute of Neurology, University College London, London WC1N 3BG, UK.

[2]CHDI Management / CHDI Foundation Inc., New York, NY 10001, USA

*Corresponding Author: gillian.bates@ucl.ac.uk

Supplementary Information

Supplementary Table S1: Recommended dilution of tissue lysate (10 mg / 300 µL) to ensure signals are within the linear range of detection for genes of interest.

Brain region	*Dilution of starting material (10 mg/300 µL)*	*Final input (µg/µL)*
Striatum	*1:3*	*11*
Cortex	*1:4-1:5*	*6.6 – 8.25*
Hippocampus	*1:3-1:5*	*6.6 – 11*
Cerebellum	*1:4-1:5*	*6.6 – 8.25*
Brainstem	*1:3-1:5*	*6.6 – 11*

Supplementary Table S2: qPCR primer / probe set sequences for the measurement of *Htt* transcripts and for the discrimination of endogenous *Htt* and mutant *Htt* in zQ175 mice.

Assay name	*ID*	*Sequence (5'-3')*	*Assay Efficiency*
(Htt)-I$_1$-pA$_1$	FW	TCCTCATCAGGCCTAAGAGCTGG	1.9
	RV	GAGACCTCCTAAAAGCATTATGTCATC	
	probe	AGTGCAGGACAGCGTGAGAGATGTG	
(Htt)-I$_1$-pA$_2$	FW	TAGCTGCTTGTGACTGGAGA	2
	RV	CCCAGAGTTGAGAGAAAGGA	
	probe	AGCTGCAAGAGAGCACAGGGC	
(Htt)- I$_1$-3'	FW	GCTTACTGCCTCTGTCCATT	2.1
	RV	TCAATCAGTCAGCTTGGAGA	
	probe	CCCAAAGGTGCTAGCCTCCA	
Htt-FL	FW	TTAGTGCCATTCATCGTAATTC	2
	RV	TGAAGTGTTTTCTTCAGAGTGG	
	probe	TCCAGGCAATTCAGTCTCGCTG	
Endogenous (WT) allele	FW	CAGGTCCGGCAGAGGAACC	1.9
	RV	TTCACACGGTCTTTCTTGGTGG	
	probe	TGCACCGACCAAAGAAGGAACTCT	

FW = forward; RV = Reverse

Supplementary Table S3: Design of the 14-plex QuantiGene assay for the detection of all *Htt* transcripts.

Transcript/gene symbol	*Transcript/gene name*	*Accession Number*	*Probe Set Region*
(Htt)-I$_1$-pA$_1$	*Huntingtin – Intron 1 - poly(A)$_1$*	*NC000071*	*521-983*
(Htt)-I$_1$-pA$_2$	*Huntingtin – Intron 1 - poly(A)$_2$*	*NC000071*	*1104-1465*
(Htt)- I$_1$-3'	*Huntingtin – Intron 1 - 3'*	*NC000071*	*16339-16922*
(Htt)-I$_3$	*Huntingtin – Intron 3*	*NC000071*	*30195-30846*
Htt-FL (Exons 50-53)	*Huntingtin coding*	*NM_010414*	*6901-7433*
(Htt-FL)-s-3'UTR	*Huntingtin –short- 3' UTR*	*NM_010414*	*9553-9993*
(Htt-FL)-m-3'UTR	*Huntingtin –mid- 3' UTR*	*NM_010414*	*10720-11120*
(Htt-FL)-l-3'UTR	*Huntingtin –long- 3' UTR*	*NM_010414*	*12640-13104*
Eif4a2	*Eukaryotic Initiation factor 4a2*	*NM_013506*	*710-1271*
Rpl13a	*Ribosomal protein L13a*	*NM_009438*	*2-467*
Ubc	*Ubiquitin C*	*NM_019639*	*113-676*
CanX	*Calnexin*	*NM_007597*	*1195-1720*
Atp5b	*ATP synthase subunit beta*	*NM_016774*	*22-406*
Ppib	*Cyclophilin B*	*NM_011149*	*207-588*

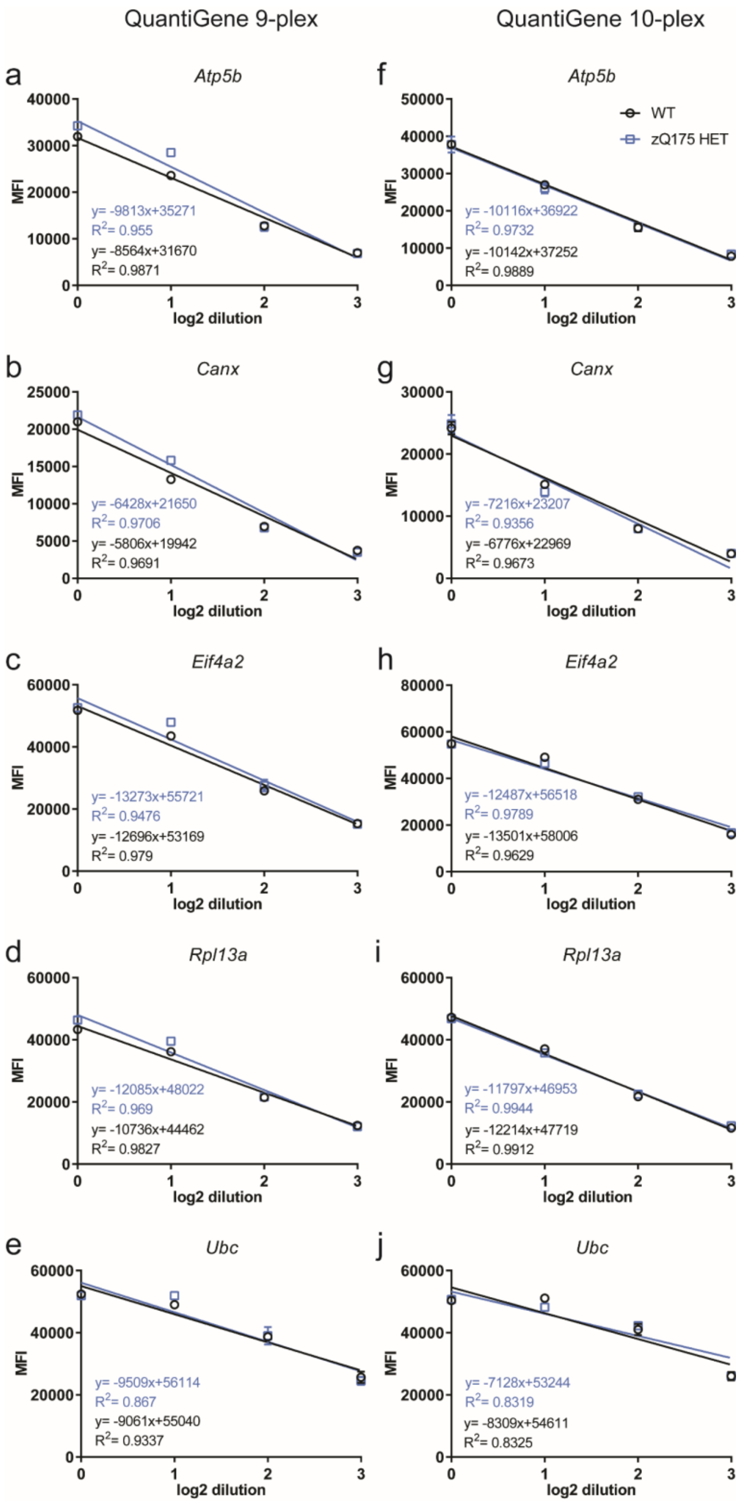

Supplementary Figure S1: Establishment and optimisation of QuantiGene *Htt* multiplex assays in striatal tissue in WT and zQ175 heterozygous mice. The MFI signals corresponding to 2-fold serial dilutions of the QuantiGene striatal lysate are shown for each housekeeping gene probe set. **(a-e)** 9-plex QuantiGene assay and **(f-j)** 10-plex QuantiGene assay. *Eif4a2* and *Ubc* reached saturation at the highest lysate concentrations for both the 9-plex and 10-plex QuantiGene assays. Data were from two technical replicates of equimolar pools of striatal samples (n = 4/genotype). Statistical analysis by linear regression. MFI = mean fluorescence intensity.

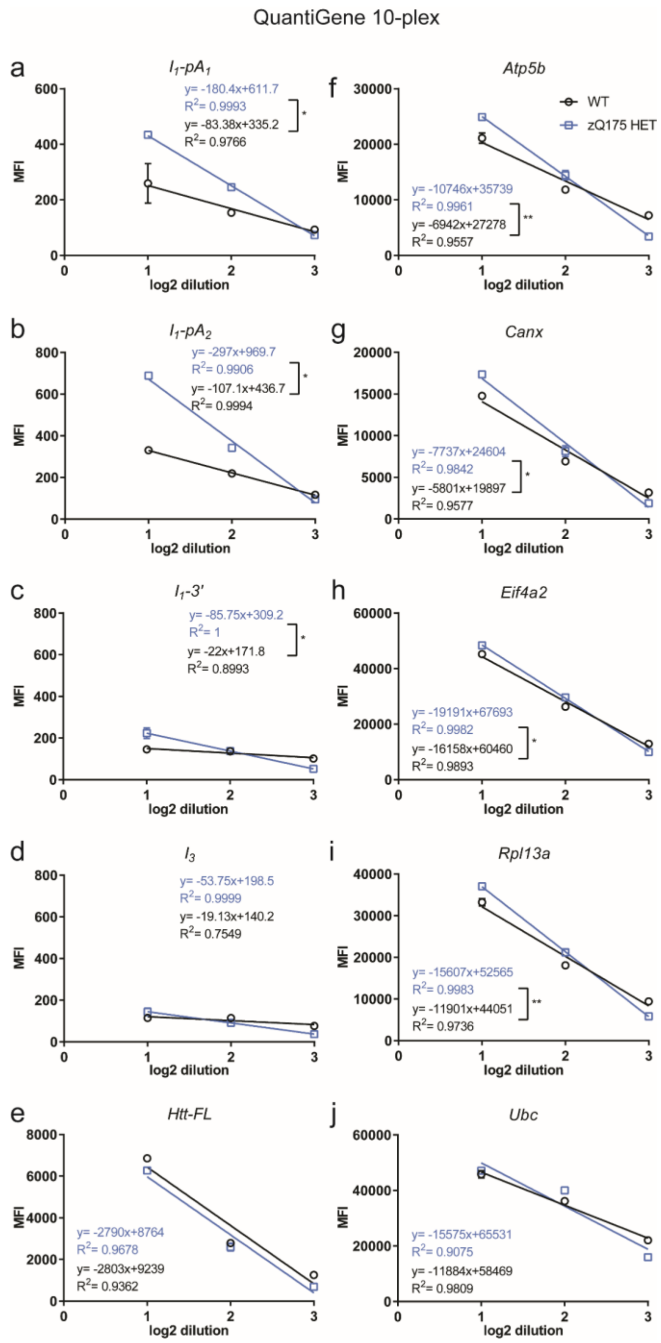

Supplementary Figure S2: Establishment and optimisation of QuantiGene *Htt* 10-plex assay in cortical tissue in WT and zQ175 heterozygous mice.

The MFI signals corresponding to 2-fold serial dilutions of the QuantiGene cortical lysate are shown for each transcript probe set. **(a-e)** *Htt* transcripts and **(f-j)** Housekeeping reference genes. **(a, b)** The linear regression slopes of the *Httexon1* transcript (I_1–pA_1 and I_1–pA_2) between the two genotypes are significantly different. **(c, d)** The I_1-*3′* and I_3 probe sets give comparable signals between WT and zQ175 samples. **(e)** *Htt-FL* probe set signals are shown. **(f-i)** The linear regression slopes for the *Atp5b*, *Canx*, *Eif4a2* and *Rpl13a* housekeeping genes are significantly different between genotypes, but the signals are comparable at the recommended dilutions (Supplementary Table S1). Data were from two technical replicates of equimolar pools of striatal samples (n = 4/genotype). Statistical analysis by linear regression, *p<0.05, **p<0.01. MFI = mean fluorescence intensity.

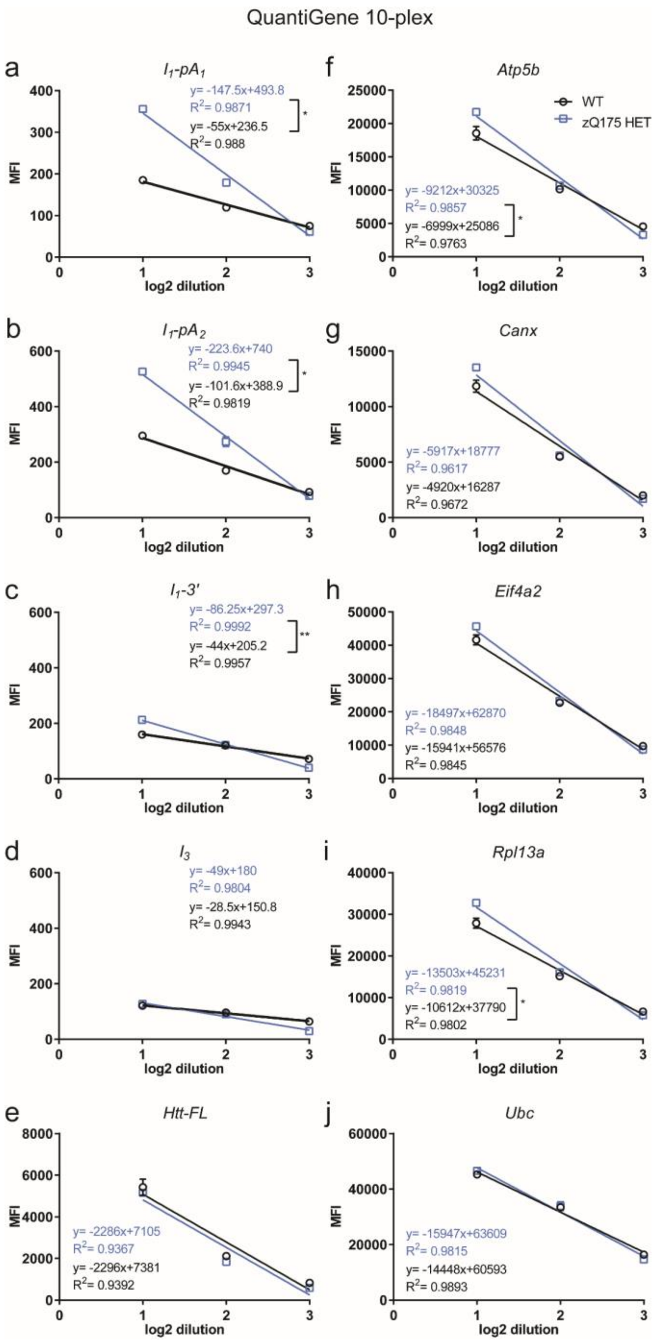

Supplementary Figure S3: Establishment and optimisation of QuantiGene *Htt* 10-plex assay in hippocampal tissue in WT and zQ175 heterozygous mice.

The MFI signals corresponding to 2-fold serial dilutions of the QuantiGene hippocampal lysate are shown for each transcript probe set. **(a-e)** *Htt* transcripts and **(f-j)** Housekeeping reference genes. **(a, b)** The linear regression slopes of the *Httexon1* transcript (I_1–pA_1 and I_1–pA_2) between the two genotypes are significantly different. **(c, d)** The I_1-*3′* and I_3 probe sets give comparable signals between WT and zQ175 samples. **(e)** *Htt-FL* probe set signals are shown. **(f, i)** The linear regression slopes for the *Atp5b* and *Rpl13a* housekeeping genes are significantly different between genotypes, but the signals are comparable at the recommended dilutions (Supplementary Table S1). Data were from two technical replicates of equimolar pools of striatal samples (n = 4/genotype). Statistical analysis by linear regression, *p<0.05, **p<0.01. MFI=mean fluorescence intensity.

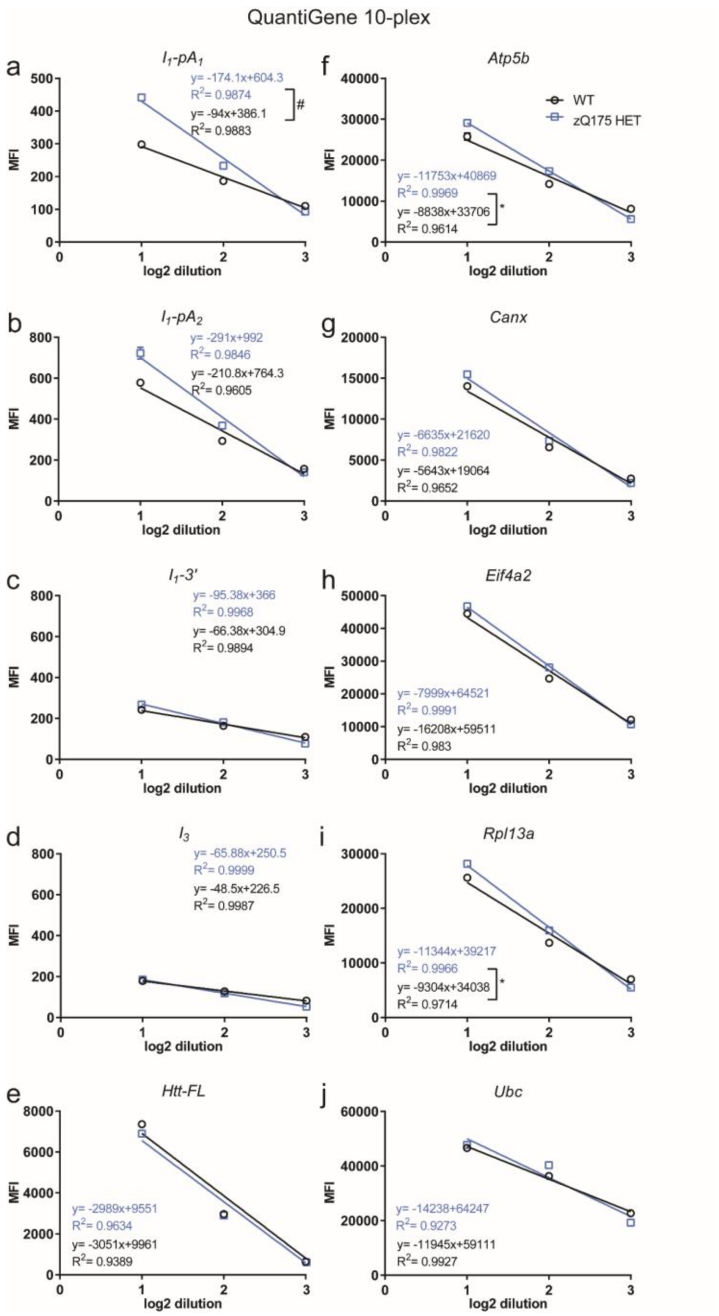

Supplementary Figure S4: Establishment and optimisation of QuantiGene *Htt* 10-plex assay in cerebellar tissue in WT and zQ175 heterozygous mice.

The MFI signals corresponding to 2-fold serial dilutions of the QuantiGene cerebellar lysate are shown for each transcript probe set. **(a-e)** *Htt* transcripts and **(f-j)** Housekeeping reference genes. **(a)** There is a trend toward a difference between the linear regression slopes between the two genotypes for the *Httexon1* transcript (I_1–pA_1) # = 0.069. **(c, d)** The I_1-*3′* and I_3 probe sets give comparable signals between WT and zQ175 samples. **(e)** *Htt-FL* probe set signals are shown. **(f, i)** The linear regression slopes for the *Atp5b* and *Rpl13a* housekeeping genes are significantly different between genotypes, but the signals are comparable at the recommended dilutions (Supplementary Table S1). Data were from two technical replicates of equimolar pools of striatal samples (n = 4/genotype). Statistical analysis by linear regression. MFI = mean fluorescence intensity.

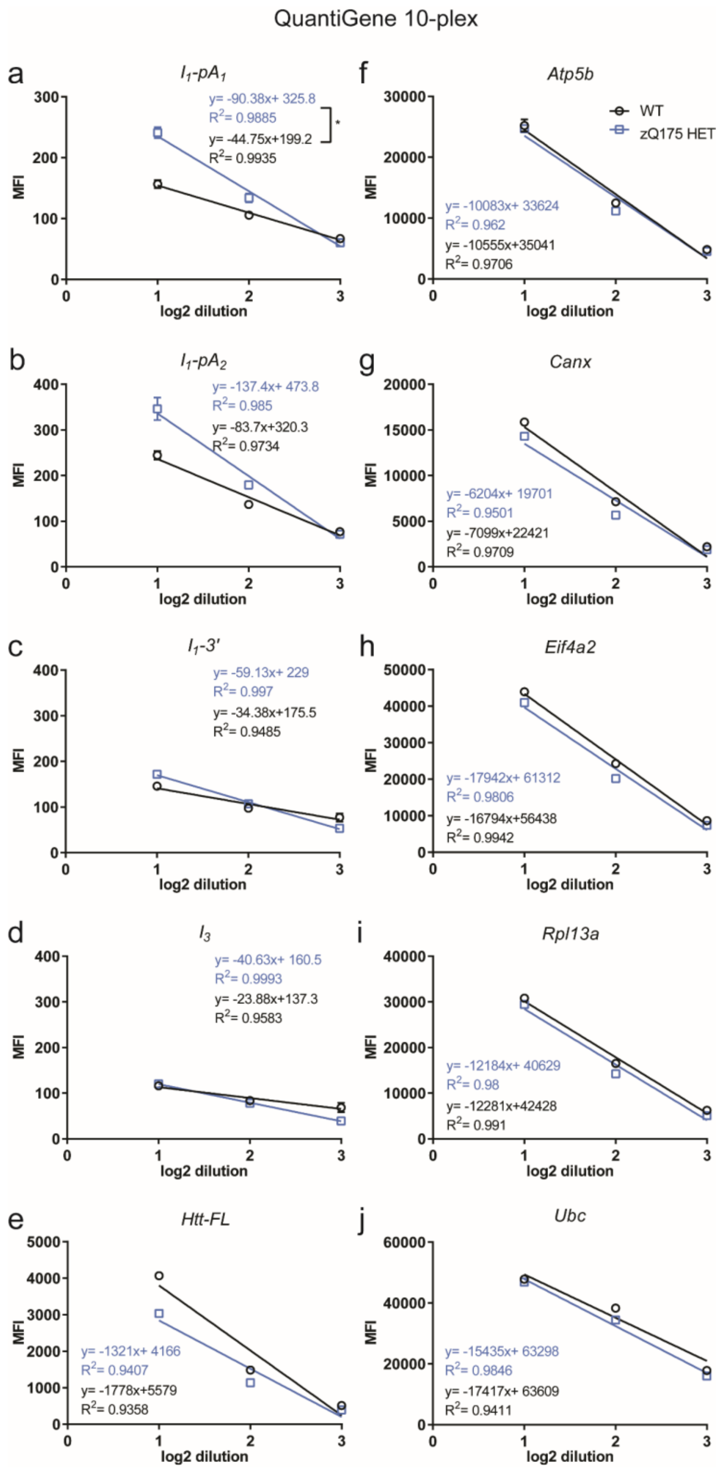

Supplementary Figure S5: Establishment and optimisation of QuantiGene *Htt* 10-plex assay in brain stem tissue in WT and zQ175 heterozygous mice. The MFI signals corresponding to 2-fold serial dilutions of the QuantiGene brain stem lysate are shown for each transcript probe set. **(a-e)** *Htt* transcripts and **(f-j)** Housekeeping reference genes. **(a)** The linear regression slope of the *Httexon1* transcript with the I_1–pA_1 probe set between the two genotypes is significantly different. **(c, d)** The I_1-$3'$ and I_3 probe sets give comparable signals between WT and zQ175 samples. **(e)** *Htt-FL* probe set signals are shown. Data were from two technical replicates of equimolar pools of striatal samples (n = 4/genotype). Statistical analysis by linear regression, *p<0.05. MFI = mean fluorescence intensity.

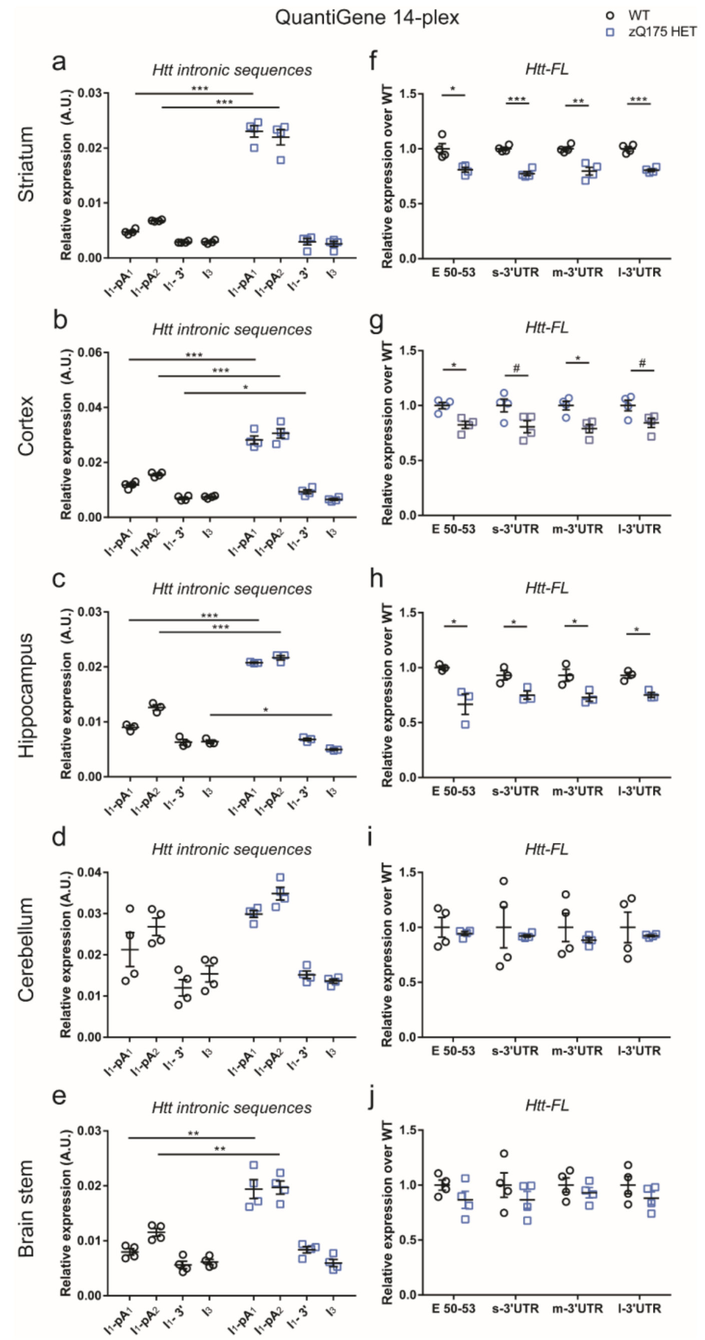

Supplementary Figure S6: Expression analysis of *Htt* transcripts in five brain regions using the custom-made 14-plex QuantiGene. The expression of intronic *Htt* transcripts is presented relative to that for housekeeping genes. **(a-e)** The *Httexon1* transcript is present in all zQ175 brain regions, but the difference between zQ175 and WT in the cerebellum did not reach statistical significance. **(f-j**) The level of the *Htt-FL* transcript is significantly decreased in the striatum, cortex and hippocampus as measured by the E50-53 and all 3'UTR probe sets. # = 0.058. Data were from n = 4/genotype samples with two technical replicates. One sample was excluded from the WT cortex and hippocampus and one from the zQ175 hippocampus, as all of the *Htt-FL* probe sets failed to show a signal above background. Statistical analysis was by multiple *t*-test with Benjamini-Hochberg correction with an alpha of 0.05, *p<0.05, **p<0.01, ***p<0.001. Tissue lysate dilutions were 1:3 for striatum and brain stem, 1:4 for cortex and cerebellum and 1:5 for hippocampus.
